# Bernese motive and goal inventory in exercise and sport: Validation of an updated version of the questionnaire

**DOI:** 10.1371/journal.pone.0193214

**Published:** 2018-02-22

**Authors:** Julia Schmid, Vanessa Gut, Achim Conzelmann, Gorden Sudeck

**Affiliations:** 1 Institute of Sport Science, University of Bern, Bern, Switzerland; 2 Institute of Sport Science, Eberhard Karls University of Tübingen, Tübingen, Germany; Leeds Beckett University, UNITED KINGDOM

## Abstract

Target group-specific intervention strategies are often called for in order to effectively promote exercise and sport. Currently, motives and goals are rarely included systematically in the design of interventions, despite the key role they play in well-being and adherence to exercise. The Bernese motive and goal inventory (BMZI) allows an individual diagnosis of motives and goals in exercise and sport in people in middle adulthood. The purpose of the present study was to elaborate on the original BMZI and to modify the questionnaire in order to improve its psychometric properties. The study is based on data from two samples (sample A: 448 employees of companies and authorities; sample B: 853 patients of a medical rehabilitation programme). We applied confirmatory factor analysis and exploratory structural equation modelling. Overall, both the original and the updated BMZI had an acceptable to good validity and a good reliability. However, the revised questionnaire had slightly better reliability. The updated BMZI consists of 23 items and covers the following motives and goals: Body/Appearance, Contact, Competition/Performance, Aesthetics, Distraction/Catharsis, Fitness and Health. It is recommended as an economical inventory for the individual diagnosis of important psychological conditions for exercise and sport.

## Introduction

### Target group-specific exercise and sport promotion

Target group-specific intervention strategies are often called for when discussing means of promoting exercise and sport activities within a population [1]. Such strategies are used to increase the relevance and salience of health information and thereby address distinct groups within the population more specifically. Furthermore, it is assumed that such target group-specific interventions are more likely to lead to changes in behaviour [2,3].

The need for target group-specific interventions to promote exercise and sport activities is a consequence of the great differences between people. Such differences are not only found between people across different stages in life, but also among people of the same age [4]. Adults differ in their exercise and sport behaviour (e.g. stage of behaviour change, level of exercise and sport), in their physical (e.g. level of fitness, health status) and psychological conditions for exercise and sport (e.g. motives and goals, self-efficacy), but also in terms of their demographic characteristics (e.g. education level, sex).

### Focus on motives and goals for exercise and sport

At present, target group-specific interventions aiming to promote exercise and sport are primarily adjusted according to their behavioural and demographic properties [1,5]. The psychological conditions for exercise and sport have rarely been taken systematically into account. However, there are indications that these features are particularly useful in identifying homogenous groups within the population [6,7].

Motives and goals are important psychological conditions for exercise and sport. Motives are a willingness to strive for certain goal conditions that endures over time and different situations [8]. Psychologists distinguish between implicit and explicit motives. Implicit motives are learned early in life and are often not consciously perceived. The explicit motives on which this paper focuses are part of an individual’s self-concept and include the values and goals that individuals ascribe to themselves. They are consciously represented and can be measured using questionnaires [9]. Personal goals, in turn, are defined as cognitive representations of events, states and processes, which an individual wants to achieve, maintain or avoid [10,11]. Explicit motives and goals are considered when offering a subjective explanation of one’s own exercise and sport behaviour (e.g. “Why do you exercise?”). Studies have shown that satisfying motives and achieving goals have a positive effect on well-being [12] and contribute to maintaining healthy behaviour [13,14].

### The original BMZI

In order to take into account motives and goals in target group-specific interventions, an instrument is needed that can measure these. One way of measuring explicit motives and goals is the Bernese Motives and Goals Inventory for exercise and sport activities (BMZI) [15]. The questionnaire focuses on the mentioned two subsets of physical activity. Exercise and sport activities are both planned, structured, and carried out during leisure time [16]. The terms differ in that exercise includes mostly activities aiming to improve or maintain physical fitness and health, whereas sport activity includes mostly traditional sports, which are associated with competition and performance.

The BMZI covers the full range of reasons for doing exercise and sport activities that may be relevant to individuals. It is based on the work of Gabler [17] and was designed for and validated in people in middle adulthood. The BMZI measures motives and goals in the following seven areas: (1) Contact, on the one hand, refers to communicating while pursuing an activity, and on the other hand, to the possibility of using sport to establish and maintain social relationships. (2) Competition/Performance describes the intention to use sport to compare oneself with others and to pursue one’s own sport-related goals. (3) Activation/Enjoyment is the aim to use sport to replenish energy and experience pleasure through exercising. Whereas Activation/Enjoyment can be considered a positive recreational component, (4) Distraction/Catharsis focuses on the negative recreational component: doing sport to take your mind off problems or everyday pressures, or to dispel negative emotions, such as stress or anger. (5) Figure/Appearance refers to having a positive effect on body weight and improving one’s physical appearance by doing sport. (6) Fitness/Health is similarly outcome-oriented, focusing on improving and maintaining one’s physical health and fitness. (7) Aesthetics constitutes aiming to experience beautiful movements during sport (e.g. harmonious movements while cross-country skiing or a successful sequence of moves when dancing).

### The need for further examination and modification of the BMZI

In the original validation studies for the BMZI, Lehnert et al. [15] judged the psychometric properties of this measure to be satisfactory to good overall. However, there were some indications that the inventory needed to be evaluated further and modified if necessary:

1. The motive area Activation/Enjoyment displayed low indicator and factor reliabilities. Additionally, its discriminant validities were problematic. This was explained by the comparatively heterogeneous items and the relatively strong connections between Activation/Enjoyment and Distraction/Catharsis as well as between Activation/Enjoyment and Aesthetics.
2. Within the motive area Fitness/Health, the item dealing with the improvement of one’s health (heal1: “especially for health reasons”) stood out critically. This item not only displayed a high degree of selectivity, but also low indicator reliability. The inter-item correlations revealed that this item was comparatively weakly connected to the two fitness items. Thus, the independence of the facets of Health and Fitness from each other should be further examined.
3. Thus far, the BMZI has primarily been used on samples from workplace settings. Lehnert et al. [15] therefore noted that the questionnaire’s usability should be explored further in the future. For example, the extent to which the inventory is also valid for specific subgroups within exercise and sport should be checked. It is currently unclear whether the BMZI is invariant across exercise levels. Lehnert et al. [15] supposed that people who are more active draw on concrete experiences when they complete the questionnaire, whereas people who are inactive refer to expectations. This different reflection process could influence the validity of the BMZI.

### Present research

Based on past empirical research by Lehnert et al. [15], the present research project set out to further elaborate on the original BMZI and, if necessary, to modify the instrument in order to improve its psychometric properties in various fields of application. Firstly, we rechecked the quality of the original questionnaire using new data. Building upon this, we secondly specifically generated new items and calculated the factorial validity and reliability of the modified questionnaire. Finally, we determined whether the original and modified versions of the BMZI could be used in different subgroups within exercise and sport. We used questionnaires both for company employees and patients undergoing medical rehabilitation programmes. Furthermore, we tested the measurement invariance across exercise levels.

The factorial validity of multidimensional questionnaires is traditionally checked using explorative (EFA) and confirmatory factor analyses (CFA). Lehnert et al. [15] used this method when developing the original BMZI. Recently, however, CFA has come under repeated criticism. Marsh et al. [18], for example, has pointed out that the basic assumption of CFA–that each item loads exclusively on a single factor–is too restrictive. In view of the need for a less restrictive measurement model, Exploratory Structural Equation Modelling (ESEM) was developed [19]. We also used this alternative procedure in the present study. ESEM combines the advantages of explorative and confirmatory factor analyses. This analytical method incorporates cross-loading, which represents the underlying structure more realistically and provides a better model fit. Additionally, ESEM allows the measurement invariance to be checked.

## Methods

### Participants and procedures

Sample A consisted of 475 employees in 3 different companies in urban areas in Switzerland, comprised of 136 women and 339 men. The employees had a mean age of 48.81 years (*SD* = 7.58; range 35–64 years). Thirty-four percent of participants indicated a degree from a university or a university of applied science as their highest level of education. Thirteen percent of the employees were physically inactive, 13% exercised for 1 to 90 min per week and 74% exercised >90 min per week. We recruited employees via e-mail and internal company health management platforms. They were invited to participate in the study by accessing the online questionnaires. Before starting the questionnaire, all employees read an informed consent form. The ethics commission of the Faculty of Humanities of the University of Bern evaluated the study design and procedures as ethically unproblematic.

Sample B is from the research project “Development of a person-oriented exercise therapy in the medical rehabilitation” [20]. This sample included 917 participants at the beginning of a medical rehabilitation programme in Germany. The sample was comprised of 397 women and 520 men. The mean age of the patients was 52.90 years (*SD* = 6.64; range from 35–64). Forty-six percent of the patients were in a rehabilitation programme for orthopaedic diseases, 24% for metabolic diseases, 20% for cardiovascular diseases and 10% for oncologic diseases. Nine percent reported a degree from a university or a university of applied science as their highest level of education. Thirty-one percent of the patients were physically inactive, 21% exercised for 1 to 90 min per week and 48% exercised >90 min per week. We recruited patients in 10 different rehabilitation clinics. Every patient received information about the study, an informed consent form and a paper-pencil questionnaire. The ethics commission of the Faculty of Behavioural and Cultural Studies of the University of Heidelberg approved the study design and procedures.

### Instruments

For assessing motives and goals in exercise and sport, we used the BMZI [15]. The original BMZI consists of 24 items and covers the following 7 motive areas: Contact (5 items), Competition/Performance (4 items), Activation/Enjoyment (3 items), Distraction/Catharsis (4 items), Figure/Appearance (3 items), Fitness/Health (3 items) and Aesthetics (2 items). To explore whether facets of Health and Fitness are better represented in 2 separate factors instead of a common factor, we added 3 new items for these facets (S1 Table). The initial question of the BMZI is “Why do you exercise/why would you exercise?” Each item is accompanied by a 5-point response scale (1 = ‘‘I strongly disagree” to 5 = “I strongly agree”).

For measurement of physical exercise and sport activities, we used the BSA Questionnaire by Fuchs et al. [21]. Participants named a maximum of 3 exercise or sport activities they had regularly engaged in within the last 4 weeks. They indicated the frequency and duration per episode in minutes for each activity. We calculated a total index value in “minutes per week”.

### Data preparation

We checked both samples for response bias and deleted 5 individuals from sample A and 23 individuals from sample B. To detect multivariate outliers, we calculated the Mahalanobis distance values as *χ*^2^ at *p* < .001 [22]. We excluded 22 multivariate outliers in sample A and 41 in sample B. We estimated missing values by means of the full information maximum likelihood (FIML) procedure [23]. Overall, the proportion of missing items was 0.8% (29 of 13440 responses) in sample A and 0.2% (213 of 25590 responses) in sample B.

### Data analyses

To test factorial validity of the BMZI, we calculated confirmatory factor analyses (CFA) and exploratory structural equation modelling (ESEM), applying a maximum likelihood estimator. We used an oblique geomin rotation. Following the recommendations of Schermelleh-Engel, Moosbrugger, and Müller [24], a good fit is indicated when *χ*^2^/df ≤ 2.00; CFI ≥ .97, and root mean square error of approximation (RMSEA) and standardized root mean square residual (SRMR) ≤ .05. For all models, measurement errors between con4 („get to know people“) and con5 („to make new friends“) were allowed to correlate. This is in line with the procedure of Lehnert et al. [15]. The correlation is theoretically grounded, as for both items the goal and motive is to get to know people *through* exercise and sport.

To assess discriminant validity, we computed the Fornell-Larcker criterion [25]. According this criterion, a multidimensional measurement instrument is discriminant valid if the average variance explained (AVE) is greater than the squared variance between the factor and other factors.

To estimate the reliability of the indicators of the BMZI, we computed squared multiple correlations (SMC). To estimate the reliability of the constructs, we calculated the composite reliability (CR; [26]) and the AVE [24]. We used SMC ≥ .50, CR ≥ .60 and AVE ≥ .50 as cut-offs for good reliabilities. We conducted all statistical analyses using Mplus 7.4 [27].

To examine whether the updated BMZI scale displayed invariance across exercise levels, we employed a sequential model testing approach via multiple group ESEM. We tested and compared 3 models: the configural model (i.e. factor-loading patterns are equal across exercise levels), the metric model (i.e. factor-loading patterns and item loadings are equal across exercise levels) and the scalar model (i.e. factor-loading patterns, item loadings and item intercepts are equal across exercise levels). Configural invariance existed if the simultaneously estimated model fit the data well [28]. Metric and scalar invariance existed if the comparative fit index (CFI) resp. RMSEA difference between the models was ≤ .010 resp. ≤ .015 [29]. To investigate measurement invariance, we divided both samples into 2 groups: (1) less active individuals who exercised 0 to 90 min per week (sample A: n = 150; sample B: n = 415) and (2) more active individuals who exercised >90 min per week (sample A: n = 298; sample B: n = 429).

## Results

### Validity and reliability of the original BMZI

The original BMZI had an acceptable to good factorial validity, both in employees and in patients (see Table 1). Fit indices of the CFA models were comparable with those of Lehnert et al. [15]. As expected, fit indices of the less restrictive ESEM models were better. The following factors were supported with the two new samples: Contact, Competition/Performance, Distraction/Catharsis, Figure/Appearance, Fitness/Health and Aesthetics. However, the Activation/Enjoyment factor was deemed problematic because items displayed relatively low loadings on the factor and cross-loadings on Distraction/Catharsis, Fitness/Health and Aesthetics (see Tables 2 and 3). The original questionnaire is discriminant valid, as all factors meet the Fornell-Larcker criterion (see Table 4).

**Table 1:** Factorial validity of the original and updated version of the BMZI.

	Sample A: emplyees (*n* = 448)						Sample B: patients (*n* = 853)					
	χ2	*df*	χ2/*df*	CFI	SRMR	RMSEA (90% CI)	χ2	*df*	χ2/*df*	CFI	SRMR	RMSEA (90% CI)
**Original BMZI**												
CFA	707.00	230	3.07	.920	.070	.068 (.062-.074)	1043.62	230	4.54	.929	.056	.064 (.060-.068)
ESEM^1^	208.41	128	1.63	.985	.016	.037 (.028-.046)	269.80	128	2.11	.987	.012	.036 (.030-.042)
**Updated BMZI**												
ESEM	170.69	112	1.52	.989	.013	.034 (.023-.044)	267.05	112	2.38	.985	.011	.040 (.034-.047)

CFA, Confirmatory factor analysis; ESEM, Exploratory structural equation modelings; CFI, Comparative Fit Index; SRMS, Standardized Root Mean Square Residual; RMSEA, Root Mean Square Error of Approximation; 90%-CI, 90-percent-confidental interval for RMSEA

^1^Due to estimation problems in sample A, we constrained (minor) negative residual of items kon1-kon5 > 0.

**Table 2:** Factor loadings of the original BMZI in sample A.

Items	Factors													
	Contact		Competition/Performance		Distraction/Catharsis		Body/Appearance		Fitness/Health		Activation/Enjoyment		Aesthetics	
	CFA	ESEM	CFA	ESEM	CFA	ESEM	CFA	ESEM	CFA	ESEM	CFA	ESEM	CFA	ESEM
con1	.91	.79												
con2	.85	.77												
con3	.78	.74												
con4	.80	.69												
con5	.79	.61												
comper1			.84	.82										
comper2			.84	.80										
comper3			.62	.47										
comper4			.61	.53										
discat1					.79	.75								
discat2					.66	.60								
discat3					.77	.64						.25		
discat4					.81	.68								
figapp1							.90	.88						
figapp2							.93	.93						
figapp3							.75	.67						.20
fit1									.83	.64		.27		
fit2									.84	.85				
heal1									.61	.58				
actenj1						.31					.77	.52		
actenj2											.68	.57		.27
actenj3										.35	.71	.37		
aes1												.23	.82	.62
aes2													.81	.86

Loadings < .20 are not presented; CFA, Confirmatory factor analysis; ESEM, Exploratory structural equation modelings.

**Table 3:** Factor loadings of the original BMZI in sample B.

Items	Factors													
	Contact		Competition/Performance		Distraction/Catharsis		Body/Appearance		Fitness/Health		Activation/Enjoyment		Aesthetics	
	CFA	ESEM	CFA	ESEM	CFA	ESEM	CFA	ESEM	CFA	ESEM	CFA	ESEM	CFA	ESEM
con1	.87	.90												
con2	.90	.92												
con3	.94	.94												
con4	.73	.61												
con5	.73	.62												
comper1			.83	.81										
comper2			.89	.88										
comper3			.67	.76										
comper4			.49	.35										
discat1					.73	.78								
discat2					.73	.76								
discat3					.87	.76						.21		
discat4					.79	.63						.21		
figapp1							.79	.82						
figapp2							.92	.92						
figapp3							.84	.84						
fit1									.71	.67				
fit2									.74	.76				
heal1									.47	.43				
actenj1						.23					.73	.52		
actenj2											.57	.37		.33
actenj3											.84	.93		
aes1													.84	.88
aes2													.80	.72

Loadings < .20 are not presented; CFA, Confirmatory factor analysis; ESEM; Exploratory structural equation modelings.

**Table 4:** Descriptive statistics, reliability and intercorrelations of the original BMZI.

	Sample	Descriptive statistics		Reliability		Intercorrelations							
		*M*	*SD*	CR	AVE	C/A	D/C	B/A	F/H	F	H	A/E	AES
Contact (C)	A	2.60	1.07	.92	.71	.37	.00	-.03	-.02	−	−	-.04	.20
	B	2.40	1.05	.90	.71	.56	.30	.12	.09	−	−	.42	.33
Competition/Performance (C/A)	A	2.18	0.89	.82	.58	−	.22	-.08	.02	−	−	.05	.33
	B	1.65	0.81	.80	.57	−	.31	.08	-.06	−	−	.39	.18
Distraction/Catharsis (D/C)	A	3.04	1.00	.86	.66	−	−	.26	.19	−	−	.41	.21
	B	3.16	1.05	.82	.61	−	−	.22	.26	−	−	.31	.48
Body/Appearance (B/A)	A	3.15	1.09	.90	.75	−	−	−	.16	−	−	.00	-.08
	B	3.69	1.14	.90	.77	−	−	−	.39	−	−	.21	.15
Fitness/Health (F/H)	A	4.34	0.57	.68	.45	−	−	−	−	−	−	.42	.21
	B	4.42	0.66	.79	.61	−	−	−	−	−	−	.18	.49
Activation/Enjoyment (A/P)	A	4.04	0.74	.73	.59	−	−	−	−	−	−	−	.41
	B	3.71	0.90	.63	.57	−	−	−	−	−	−	−	.42
Aesthetics (AES)	A	3.26	1.23	.80	.68	−	−	−	−	−	−	−	−
	B	2.57	1.16	.78	.69		−	−	−	−	−	−	−

CR, Composite reliability; AVE, Average variance explained.

The majority of indicator reliabilities of the original BMZI were good. Yet, single items of Activation/Enjoyment (actenj1: SMC_sample A_ = .48; actenj2: SMC_sample A_ = .42), Fitness/Health (fit1: SMC_sample A_ = .48; heal1: SMC_sample A_ = .25) and Competition/Performance (comper3: SMC_sample B_ = .45; comper4: SMC_sample A_ = .32, SMC_sample B_ = .39) stood out with problematically low SMC. The factorial reliabilities of the original BMZI were good overall, with the exception of Fitness/Health, which had a low AVE = .45 among employees.

### Brief discussion

Overall, findings of the first analyses supported 6 out of 7 motives and goals. To improve the simple factor structure, we removed all 3 items, tapping Activation/Enjoyment (actenj1: “to relax”, actenj2: “Primarily out of joy of movement”, actenj3: “to replenish new energy”) for further analyses. Furthermore, we excluded comper4 (“for the thrill”) from the factor Competition/Performance. The item had a relatively low primary loading and low indicator reliability in both employees and patients. The item was removed already in other validation studies in older adults [30]. Concerning the motive and goal Fitness/Health, the findings confirm the problematic psychometric quality of the item heal1, but only in employees. To explore options for further improvements of psychometric properties, we added 3 new items to distinguish between the factors of fitness and health instead of a common health/fitness factor.

### Validity and reliability of the updated BMZI

The updated BMZI had a good factorial validity, both in employees and patients (see Table 1). The data followed the expected factor structure (see Table 5). The following 7 factors form the updated BMZI: Contact, Competition/Performance, Distraction/Catharsis, Figure/Appearance, Aesthetics, Fitness and Health. However, when considering factor loadings and factor correlations it is noticeable that Fitness is more strongly related with Health in patients than in employees. The updated questionnaire is discriminant valid, since all 7 factors meet the Fornell-Larcker criterion (see Table 4 and Table 6).

**Table 5:** Standardized factor loadings (ESEM) of the updated BMZI in sample A and B.

Items	Factors													
	Contact		Competition/Performance		Distraction/Catharsis		Body/Appearance		Fitness		Health		Aesthetics	
	Sample A	Sample B	Sample A	Sample B	Sample A	Sample B	Sample A	Sample B	Sample A	Sample B	Sample A	Sample B	Sample A	Sample B
con1	.88	.80												
con2	.90	.79												
con3	.91	.75												
con4	.60	.67	.22											
con5	.60	.58	.21	.20										.21
comper1			.79	.88										
comper2			.84	.75										
comper3			.70	.46										.21
discat1					.77	.78								
discat2					.70	.60								
discat3					.81	.71								
discat4					.69	.75								
figapp1							.81	.90					.22	
figapp2							.90	.88						
figapp3							.80	.68						
fit1									.65	.73				
fit2									.70	.62		.23		
fit3*									.70	.63	.22	.26		
heal1											.67	.65		
heal2*											.65	.72		
heal3*										.25	.73	.56		
aes1													.78	.76
aes2													.84	.80

Loadings < .20 are not presented; CFA, Confirmatory factor analysis; ESEM, Exploratory structural equation modelling.

**Table 6:** Descriptive statistics, reliability and intercorrelations of the updated BMZI.

	Sample	Descriptive statistics		Reliability		Intercorrelations							
		*M*	*SD*	CR	AVE	C/A	D/C	B/A	F/H	F	H	A/E	AES
Contact (C)	A	2.60	1.07	.92	.72	.25	-.03	-.01	−	-.05	.00	−	.15
	B	2.40	1.05	.90	.70	.36	.15	.04	−	.02	.03	−	.31
Competition/Performance (C/A)	A	2.31	1.00	.85	.67	−	.15	-.06	−	-.01	-.04	−	.22
	B	1.70	0.85	.80	.63	−	.20	.05	−	-.02	-.11	−	.31
Distraction/Catharsis (D/C)	A	3.04	1.00	.87	.65	−	−	.16	−	.11	.14	−	.20
	B	3.16	1.05	.84	.60	−	−	.11	−	.14	.13	−	.25
Body/Appearance (B/A)	A	3.15	1.09	.89	.75	−	−	−	−	.08	.18	−	-.04
	B	3.69	1.14	.89	.76	−	−	−	−	.22	.27	−	.15
Fitness (F)	A	4.43	0.58	.77	.59	−	−	−	−	−	.42	−	.15
	B	4.38	0.70	.82	.71	−	−	−	−	−	.60	−	.16
Health (H)	A	4.00	0.79	.77	.58	−	−	−	−	−	−	−	.03
	B	4.46	0.67	.77	.64	−	−	−	−	−	−	−	.13
Aesthetics (AES)	A	3.26	1.23	.80	.68	−	−	−	−	−	−	−	−
	B	2.57	1.16	.79	.67	−	−	−	−	−	−	−	−

CR, Composite reliability; AVE, Average variance explained.

The total number of low indicator reliabilities could be reduced within the updated questionnaire. Nonetheless, items of Fitness and Health (fit1: SMC_sample A_ = .49, heal1: SMC_sample B_ = .40), Distraction/Catharsis (discat2: SMC_sample B_ = .46) and Competition/Performance (comper3: SMC_sample B_ = .44) had SMCs under the recommended criterion of .50. However, all factorial reliabilities of the updated BMZI were good.

### Measurement invariance of the updated BMZI

Configural invariance was demonstrated for the updated BMZI, since both configural models fitted the data well (see Table 7). Metric and scalar invariance were demonstrated, as ΔCFI resp. ΔRMSEA between the models were ≤ .010 resp. ≤ .015. Overall, multiple group ESEM suggested that the factor-loading patterns, item loadings and item intercepts are equal across exercise levels, both in employees and in patients. This means that the updated BMZI measures the same constructs in the same way across both less and more active people.

**Table 7:** Analysis of invariance of the updated version of the BMZI across exercise levels.

	Sample A: employees (*n* = 448)							Sample B: patients (*n* = 853)						
	χ2	*df*	CFI	SRMR	RMSEA (90% CI)	ΔCFI	ΔRMSEA	χ2	*df*	CFI	SRMR	RMSEA (90% CI)	ΔCFI	ΔRMSEA
Less active group (independent ESEM)	17.47	112	.962	.022	.068 (.046-.087)	−	−	192.32	112	.984	.013	.042 (.031-.051)	−	−
More active group (independent ESEM)	15.82	112	.990	.015	.032 (.017-.045)	−	−	179.37	112	.987	.013	.037 (.027-.047)	−	−
Configural model	294.12	224	.987	.017	.037 (.024-.049)	−	−	371.59	224	.986	.013	.040 (.032-.047)	−	−
Metric model	407.16	336	.987	.031	.035 (.018- .041)	.000	.002	512.12	336	.983	.026	.035 (.029-.041)	.003	.005
Scalar model	483.02	359	.977	.052	.039 (.030-.048)	.010	-.002	551.61	359	.981	.031	.036 (.030-.041)	.005	.004

CFA, Confirmatory factor analysis; ESEM, Exploratory structural equation modelings; CFI, Comparative Fit Index; TLI, Tucker-Lewis Index; SRMR, Standardized Root Mean Square Residual; RMSEA, Root Mean Square Error of Approximation; 90%-CI, 90-percent-confidental interval for RMSEA.

### Overall discussion

The purpose of the present study was to elaborate the original BMZI and to modify the questionnaire in order to improve its psychometric properties. Results confirmed the acceptable to good factorial validity of the original BMZI, especially for patients. However, as Activation/Enjoyment had critical loading structures, we have deleted the items of this motive and goal in an optimised version of the questionnaire. The original BMZI had a good discriminant validity as well as acceptable to good composite and indicator reliability. The updated BMZI had a good factorial validity. It covers the following motives and goals: Body/Appearance, Contact, Competition/Performance, Aesthetics, Distraction/Catharsis and Fitness and Health. The updated BMZI had good discriminant validity and a slightly better reliability than the original questionnaire. Furthermore, analysis showed that Health is significantly related to Fitness, especially in patients. Presumably because of their somatic diseases, patients associate their fitness more directly with their health issues. The questionnaire displayed invariance across exercise levels. Thus, the updated BMZI may be used in both less and more active individuals and to conduct group comparisons.

In conclusion, the results show that the updated BMZI can be used both in non-clinical settings (e.g. workplace health promotion, training programmes in fitness centres) with mostly healthy people as well as in clinical settings (e.g. exercise therapy) with patients. For the latter field of application, it must be kept in mind that the BMZI covers the motive and goal health in a general manner. Probably, for people with certain diseases and symptoms, more specific health goals are more appropriate.

The original and updated BMZIs were designed for and validated in people in middle adulthood (35–64 years). From a theoretical point of view, it is not appropriate to use the same questionnaire in younger and older people. Individuals modify their personal goals over time because their developmental tasks, role transitions and life situations change across their life span [31]. Schmid, Molinari, Lehnert, Sudeck and Conzelmann [30] previously evaluated an adapted BMZI for people over 65 years of age. Future works should investigate how existing motives and goals in exercise and sport need to be supplemented or adjusted for people under 35 years of age.

The revised questionnaire can be connected with self-determination theory [32]. By removing Activation/Enjoyment–which represents an intrinsic behavioural regulation–the updated BMZI more strictly targets goal content and more clearly differentiates between goal content and behavioural regulation of goals [33]. This conceptual differentiation has already been taken into account in an English goal content questionnaire on exercise context [34].

Future psychometric investigations of the BMZI in middle adulthood should address some of the limitations of the present study: (1) We did not select our samples randomly. Particularly, the sample of employees is probably not representative compared with the general (healthy) population. The appropriate next step would be to perform a similar series of analyses with more diverse samples with respect to gender and educational level. The representative samples could furthermore be used to determine standardised norms of the updated BMZI. (2) When testing the measurement invariance across activity levels, we did not compare inactive people with active people, as suggested by Lehnert et al. [15]. Because group sizes would have been too small (especially in sample A), we set the cut-off value to 90 min of exercise and sport activities per week. Although, the results presented indicate that activity does not influence the measurement, further research with totally inactive people is needed. (3) Our findings support the factorial and discriminant validity as well as the indicator and composite reliability of the updated BMZI. However, to date, the test–retest reliability of the questionnaire remains unknown. Future studies should investigate whether the updated BMZI is consistent over time.

We assume that motives and goals are important psychological conditions for exercise and sport. They influence self-determination, well-being, and behaviour [35]. However, what remains unclear is the *relative* importance of motives and goals to predict exercise and sport behaviour. Subsequent research should address how much motives and goals influence behaviour in comparison to other psychological (e.g. self-efficacy) or physiological constructs (e.g. health status).

In summary, we recommend the BMZI as an economical inventory for the individual diagnosis of important psychological conditions for exercise and sport in middle adulthood. The updated questionnaire helps to design target group-specific interventions to promote exercise and sport. In a first step, groups with distinct profiles of motives and goals should be identified. To collect data, the questionnaire can be used as paper-pencil or online test. To define homogeneous groups within population, cluster analysis or latent profile analysis is recommended. The identified groups can be characterized with further physiological, psychological and demographic variables (e.g. level of fitness, stage of behaviour change, sex). In doing so, a holistic picture of the target group can be achieved. In a second step, tailored interventions should be developed. It must be specified which kinds of exercise and sport is suitable for which group of people to achieve their individual goals. Sudeck and Conzelmann [12] as well as Krauss, Katzmarek, Rieger, and Sudeck [36] gave an example of target-group specific interventions based on motives and goals. It is expected that target-group specific interventions are more likely to lead to long-term changes in behaviour. Thereby, health benefits of exercise and sport should be improved, which in turn contributes to cost-effectiveness of interventions.

## Supporting information

S1 Table German and English version of the original and updated BMZI. (DOCX)

S2 Table Descriptive statistics of the items of the original and updated BMZI. Items with # were part of the updated version of the BMZI; items with * were newly phrased for the updated version of the BMZI.
(DOCX)

## References

[1] World Health Organization. Physical activity strategy for the WHO European Region 2016–2025 World Health Organization Vilnius; 2015.

[2] Hawkins RP, Kreuter M, Resnicow K, Fishbein M, Dijkstra A. Understanding tailoring in communicating about health. Health Education Research. 2008;23(3):454–66. doi: 10.1093/her/cyn004 18349033 10.1093/her/cyn004 PMC3171505

[3] Rimer BK, Kreuter MW. Advancing tailored health communication: A persuasion and message effects perspective. Journal of Communication. 2006;56(s1):S184–S201. doi: 10.1111/j.1460-2466.2006.00289.x

[4] Chamorro-Premuzic T, von Stumm S, Furnham A, editors. The Wiley-Blackwell Handbook of Individual Differences 1st ed. Wiley-Blackwell handbooks in personality and individual differences. Oxford: Wiley-Blackwell; 2011. 632 p.

[5] Noar SM, Benac CN, Harris MS. Does tailoring matter? Meta-analytic review of tailored print health behavior change interventions. Psychological Bulletin. 2007;133(4):673–93. doi: 10.1037/0033-2909.133.4.673 . 17592961 10.1037/0033-2909.133.4.673

[6] Boslaugh SE, Kreuter MW, Nicholson RA, Naleid K. Comparing demographic, health status and psychosocial strategies of audience segmentation to promote physical activity. Health Education Research. 2005;20(4):430–8. doi: 10.1093/her/cyg138 15572439 10.1093/her/cyg138

[7] Havitz ME, Kaczynski AT, Mannell RC. Exploring relationships between physical activity, leisure involvement, self-efficacy, and motivation via participant segmentation. Leisure Sciences. 2013;35(1):45–62. doi: 10.1080/01490400.2013.739890

[8] McClelland DC. Human motivation Cambridge, New York: Cambridge University Press; 1987 xii, 663.

[9] Brunstein J. Implicit and explicit motives In: Heckhausen J, Heckhausen H, editors. Motivation and action. 2nd ed. New York: Cambridge University Press; 2010 p. 227–46.

[10] Austin JT, Vancouver JB. Goal constructs in psychology: Structure, process, and content. Psychological Bulletin. 1996;120(3):338–75.

[11] Emmons RA. Striving and feeling. Personal goals and subjective well-being In: Gollwitzer PM, Bargh JA, editors. The psychology of action: Linking cognition and motivation to behavior. New York: Guilford Press; 1996 p. 313–37.

[12] Sudeck G, Conzelmann A. Motivbasierte Passung von Sportprogrammen. Explizite Motive und Ziele als Moderatoren von Befindlichkeitsveränderungen durch sportliche Aktivität [Motive-based tailoring of sports programmes. Explicit motives and goals as moderators of mood changes through sports activities]. Sportwissenschaft. 2011;41:175–89. doi: 10.1007/s12662-011-0194-8

[13] Klusmann V, Musculus L, Sproesser G, Renner B. Fulfilled emotional outcome expectancies enable successful adoption and maintenance of physical activity. Frontiers in Psychology. 2016;6(121):157 doi: 10.3389/fpsyg.2015.01990 . 26779095 10.3389/fpsyg.2015.01990 PMC4701923

[14] Rothman AJ. Toward a theory-based analysis of behavioral maintenance. Health Psychology. 2000;19(1):64–9. 10709949 10.1037/0278-6133.19.suppl1.64

[15] Lehnert K, Sudeck G, Conzelmann A. BMZI–Berner Motiv- und Zielinventar im Freizeit- und Gesundheitssport [BMZI–Bernese Motive and Goal Inventory in leisure and health sports]. Diagnostica. 2011;57(3):146–59. doi: 10.1026/0012-1924/a000043.de

[16] Straht SJ, Kaminsky LA, Ainsworth BE, Ekelund U, Freedson PS, Gary RA et al Guide to the assessment of physical activity: Clinical and research application. A scientific statement from the American Heart Association. Circulation. 2013;128:2259–2279. doi: 10.1161/01.cir.0000435708.67487.da 24126387 10.1161/01.cir.0000435708.67487.da

[17] Gabler H. Motive im Sport [Motives in exercise and sport] Schorndorf: Hofmann; 2002.

[18] Marsh H, Muthén B, Asparouhov T, Lüdtke O, Robitzsch A, Morin AJS, et al Exploratory structural equation modeling, integrating CFA and EFA: Application to students' evaluations of university teaching. Structural Equation Modeling: A Multidisciplinary Journal. 2009;16(3):439–76. doi: 10.1080/10705510903008220

[19] Asparouhov T, Muthén B. Exploratory structural equation modeling. Structural Equation Modeling: A Multidisciplinary Journal. 2009;16(3):397–438. doi: 10.1080/10705510903008204

[20] Huber G, Sudeck G. Entwicklung einer person-orientierten Bewegungstherapie in der medizinischen Rehabilitation. [Development of a person-oriented exercise therapy in medical rehabilitation]. Abschlussbericht; 2014.

[21] Fuchs R, Klaperski S, Gerber M, Seelig H. Messung der Bewegungs- und Sportaktivität mit dem BSA-Fragebogen: Eine methodische Zwischenbilanz [Measurement of physical activity and sport activity with the BSA questionnaire]. Zeitschrift für Gesundheitspsychologie. 2015;23(2):60–76. doi: 10.1026/0943-8149/a000137

[22] Tabachnik BG, Fidell LS. Using multivariate statistics 6th ed. Boston: Pearson Education; 2013.

[23] Little RJA, Rubin DB. Statistical analysis with missing data 3rd ed Chichester: Wiley; 2012.

[24] Schermelleh-Engel K, Moosbrugger H, Müller H. Evaluating the fit of structural equation models: Tests of significance and descriptive goodness-of-fit Measures. Methods of Psychological Research Online. 2003;8:23–74.

[25] Fornell C, Larcker DF. Evaluation structural equation models with unobservable variables and measurement error. Journal of Marketing Research. 1981;18:39–50.

[26] Bagozzi RP, Yi Y. Specification, evaluation, and interpretation of structural equation models. Journal of the Academy of Marketing Science. 2012;40:8–34.

[27] Muthén LK, Muthén BO. Mplus User’s Guide Muthén & Muthén. 7th ed. Los Angeles; 1998–2015.

[28] Byrne BM. Structural equation modeling with Mplus: Basic concepts, applications, and programming Multivariate applications series. New York: Routledge; 2011.

[29] Chen FF. Sensitivity of goodness of fit indexes to lack of measurement invariance. Structural Equation Modeling: A Multidisciplinary Journal. 2007;14(3):464–504. doi: 10.1080/10705510701301834

[30] Schmid J, Molinari V, Lehnert K, Sudeck G, Conzelmann A. BMZI-HEA. Adaptation des Berner Motiv- und Zielinventars im Freizeit- und Gesundheitssport für Menschen im höheren Erwachsenenalter [BMZI-HEA. Adapting the Bernese Motive and Goal Inventory in Leisure and Health Sports for people in late adulthood]. Zeitschrift für Gesundheitspsychologie. 2014;22(3):104–17. doi: 10.1026/0943-8149/a000119 de.

[31] Brunstein JC, Schultheiss OC, Maier GW. Pursuit of personal goals: A motivational approach to well-being and life adjustment In: Brandtstädter J, Lerner RM, editors. Action & self-development: Theory and research through the life span. Thousand Oaks: SAGE; 1999 169–196.

[32] Ryan RM, Deci EL. Overview of self-determination theory: An organismic dialectical perspective In: Deci EL, Ryan RM, editors. Handbook of self-determination research. Rochester, NY: University of Rochester Press; 2002 p. 3–33.

[33] Deci EL, Ryan RM. The "What" and "Why" of goal pursuits: Human needs and the self-determination of behavior. Psychological Inquiry. 2000;11:227–68.

[34] Sebire SJ, Standage M, Vansteenkiste M. Development and validation of the goal content for exercise questionnaire. Journal of Sport & Exercise Psychology. 2008;30:353–77. 18723897 10.1123/jsep.30.4.353

[35] Gunnell KE, Crocker PRE, Mack DE, Wilson PM, Zumbo BD. Goal contents, motivation, psychological need satisfaction, well-being and physical activity: A test of self-determination theory over 6 months. Psychology of Sport and Exercise. 2014; 15:19–29. http://dx.doi.org/10.1016/j.psychsport.2013.08.005

[36] Krauss I, Katzmarek U, Rieger MA, Sudeck G. Motives for physical exercise participation as a basis for the development of patient-oriented exercise interventions in osteoarthritis: A cross-sectional study. European Journal of Physical and Rehabilitation Medicine. 2017 doi: 10.23736/S1973-9087.17.04482–3 . 28215059 10.23736/S1973-9087.17.04482-3

