# Supplementary material for: Bernese motive and goal inventory in exercise and sport: Validation of an updated version of the questionnaire
Source: PLoS One. 2018 Feb 22;13(2):e0193214. doi: 10.1371/journal.pone.0193214 (PMC5823435; doi:10.1371/journal.pone.0193214)
Supplement: S1 Table — (DOCX) [file pone.0193214.s001.docx]

**Supporting information S1**

**Table. German and English version of the original and updated BMZI**

| **Item** | **German version: Warum treiben Sie Sport?/Warum würden Sie Sport treiben?** | **English translation: Why do you exercise?/Why would you exercise?** |
| --- | --- | --- |
| discat1 | Um Ärger und Gereiztheit abzubauen. | To reduce anger and tension. |
| discat2 | Weil ich mich so von anderen Problemen ablenke. | To distract myself from other problems. |
| discat3 | Um Stress abzubauen. | To reduce stress. |
| discat4 | Um meine Gedanken im Kopf zu ordnen. | To organize my thoughts. |
| fit1 | Um mich in körperlich guter Verfassung zu halten. | To keep myself in good physical shape. |
| fit2 | Vor allem um fit zu sein. | Primarily to be fit. |
| fit3 | Vor allem um etwas für meine körperliche Fitness zu tun. | Primarily to do something for my physical fitness. |
| heal1 | Vor allem aus gesundheitlichen Gründen. | Primarily for health reasons. |
| heal2 | Vor allem um meinen Gesundheitszustand zu verbessern. | Primarily to improve my state of health |
| heal3 | Um körperlichen Beschwerden entgegenzuwirken. | To work against physical health problems. |
| comper1 | Weil ich im Wettkampf aufblühe. | Because I thrive on competition. |
| comper2 | Um mich mit anderen zu messen. | To compete with others |
| comper3 | Um sportliche Ziele zu erreichen. | To achieve my exercise goals. |
| aes1 | Weil es mir Freude bereitet, die Schönheit der menschlichen Bewegung im Sport zu erleben. | For enjoyment of beautiful movements in exercise. |
| aes2 | Weil Sport mir die Möglichkeit für schöne Bewegungen bietet. | Because exercise offers me the possibility for beautiful movements. |
| con1 | Um mit anderen gesellig zusammen zu sein. | To be social with others. |
| con2 | Um etwas in einer Gruppe zu unternehmen. | To do something in a group. |
| con3 | Um dabei Freunde/Bekannte zu treffen. | To meet friends and acquaintances. |
| con4 | Um dadurch Menschen kennen zu lernen. | To get to know people. |
| con5 | Um durch den Sport neue Freunde zu gewinnen. | To make new friends through exercise. |
| figapp1 | Um abzunehmen. | To lose weight. |
| figapp2 | Um mein Gewicht zu regulieren. | To regulate my weight. |
| figapp3 | Wegen meiner Figur. | Because of my body shape. |
