# Supplementary material for: Bernese motive and goal inventory in exercise and sport: Validation of an updated version of the questionnaire
Source: PLoS One. 2018 Feb 22;13(2):e0193214. doi: 10.1371/journal.pone.0193214 (PMC5823435; doi:10.1371/journal.pone.0193214)
Supplement: S2 Table — Items with # were part of the updated version of the BMZI; items with * were newly phrased for the updated version of the BMZI. (DOCX) [file pone.0193214.s002.docx]

**Supporting information S2**

**Table. Descriptive statistics of the items of the original and updated BMZI.**

Items with # were part of the updated version of the BMZI; items with * were newly phrased for the updated version of the BMZI.

| **Motive and goal** | **Item** | **Sample A: employees (n=448)** | | | | **Sample B: patients (n=853)** | | | |
| --- | --- | --- | --- | --- | --- | --- | --- | --- | --- |
|  |  | ***M*** | ***SD*** | ***S*** | ***E*** | ***M*** | ***SD*** | ***S*** | ***E*** |
| Distraction/Catharsis | discat1# | 2.79 | 1.24 | -.05 | -1.12 | 2.92 | 1.31 | -.05 | -1.10 |
|  | discat2# | 2.66 | 1.23 | .21 | -.98 | 2.94 | 1.34 | -.02 | -1.15 |
|  | discat3# | 3.33 | 1.14 | -.37 | -.62 | 3.72 | 1.18 | -.71 | -.31 |
|  | discat4# | 3.39 | 1.13 | -.41 | -.52 | 3.04 | 1.25 | -.17 | -.92 |
| Fitness | fit1# | 4.59 | .60 | -1.35 | 1.67 | 4.36 | .77 | -1.15 | 1.23 |
|  | fit2# | 4.27 | .76 | -.93 | .84 | 4.39 | .79 | -1.32 | 1.71 |
|  | fit3#* | 4.43 | .68 | -.96 | .39 | 4.43 | .77 | -1.47 | 2.44 |
| Health | heal1# | 4.15 | .84 | -.83 | .37 | 4.52 | .78 | -1.82 | 3.47 |
|  | heal2#* | 3.98 | .96 | -.80 | .19 | 4.54 | .73 | -1.78 | 3.53 |
|  | heal3#* | 3.87 | 1.02 | -.68 | -.17 | 4.33 | .82 | -1.24 | 1.46 |
| Competition/Performance | comper1# | 1.99 | 1.19 | .93 | -.30 | 1.48 | .89 | 1.98 | 3.37 |
|  | comper2# | 2.10 | 1.08 | .63 | -.59 | 1.57 | .93 | 1.69 | 2.27 |
|  | comper3# | 2.86 | 1.18 | .04 | -.83 | 2.01 | 1.16 | .90 | -.21 |
|  | comper4 | 1.79 | .99 | 1.14 | .43 | 1.47 | .91 | 2.19 | 4.44 |
| Activation/Enjoyment | actenj1 | 3.90 | .98 | -.85 | .51 | 3.56 | 1.13 | -.55 | -.27 |
|  | actenj2 | 4.23 | .84 | -1.14 | 1.39 | 3.46 | 1.16 | -.39 | -.57 |
|  | actenj3 | 3.99 | .89 | -.89 | .95 | 4.09 | .98 | -1.01 | .68 |
| Aesthetics | aes1# | 3.39 | 1.39 | -.48 | -1.04 | 2.36 | 1.20 | .52 | -.68 |
|  | aes2# | 3.14 | 1.30 | -.27 | -1.05 | 2.58 | 1.28 | .32 | -.93 |
| Contact | con1# | 2.87 | 1.36 | -.01 | -1.24 | 2.55 | 1.26 | .33 | -.89 |
|  | con2# | 2.76 | 1.31 | .15 | -1.14 | 2.38 | 1.19 | .46 | -.70 |
|  | con3# | 2.76 | 1.27 | .11 | -1.06 | 2.14 | 1.13 | .73 | -.27 |
|  | con4# | 2.31 | 1.06 | .36 | -.75 | 3.59 | 1.31 | -.61 | -.72 |
|  | con5# | 2.31 | 1.11 | .44 | -.72 | 3.82 | 1.22 | -.92 | -.02 |
| Figure/Appearance | figapp1# | 2.81 | 1.27 | .08 | -1.05 | 3.65 | 1.23 | -.65 | -.47 |
|  | figapp2# | 3.35 | 1.19 | -.42 | -.66 | 2.36 | 1.20 | .52 | -.68 |
|  | figapp3# | 3.30 | 1.15 | -.26 | -.71 | 2.58 | 1.28 | .32 | -.93 |
